# Editorial: Biofunctional materials and coatings for orthopaedic and dental applications

**DOI:** 10.3389/fbioe.2023.1203815

**Published:** 2023-04-21

**Authors:** Ana Marina Ferreira, Piergiorgio Gentile

**Affiliations:** School of Engineering, Newcastle University, Newcastle upon Tyne, United Kingdom

**Keywords:** biofunctional materials, surface functionalisation, coatings, bone repair, dental restoration

This Research Topic includes an excellent number of studies on the design and implementation of biofunctional materials and coatings in orthopaedic and dental applications. A review of the state-of-the-art in the field of the use of biomaterials for osteogenesis advances in the development of antibacterial coatings for orthopaedic applications, the use of dual drug delivery platforms for bone tissue engineering, and a focus on the potentials of marine-derived fucoidan in bone tissue engineering are all included in this Research Topic. Furthermore, a number of original research works on the development and application of bioactive coatings of implants using novel approaches such as Cu doped TiO_2_ coated implant prepared by micro-arc oxidation by Kang et al. and titania-modified zirconia implant prepared by ultraviolet irradiation by Tang et al. The synthesis of advanced blends of calcium orthophosphate powders and mesoporous silicon particles obtained from recycled oyster shells is also described as a sustainable approach for use in bone tissue engineering strategies. Overall, this research subject provides new perspectives and viewpoints on tackling existing challenges in orthopaedic and dental applications related to osseointegration of materials, bacteria colonization, and biofilm formation.

In more detail, Wang et al. conducted an exhaustive bibliometric analysis of 1,523 papers on biomaterials research in osteogenesis between the years 2000 and 2021, highlighting the research community’s growing interest in advancing on this subject. The authors anticipate that research on the mechanisms of osteogenesis in biomaterials, bone tissue engineering for biomaterial osteogenesis, and regenerative medicine for biomaterial osteogenesis will continue to expand as research hotspots in the future, and they advise on the need for international synergies to advance in the field of osteogenesis biomaterials research. In this line, the use of bioactive materials, such as carbonated hydroxyapatite and analogues, have been widely exploited by the scientific community due to their good surface bioactivity and biocompatibility, and their ability to interact with water which improves protein adhesion and enhances cellular bio functions (e.g., cell adhesion, proliferation, osteogenic differentiation, and proangiogenic growth factor production). The research work by Richard et al. on the synthesis of biphasic calcium phosphates by the solid-state chemical reaction method of recycled oyster shells showcases a sustainable approach to scale up the production of mechanically mixed blends of calcium orthophosphate powders and mesoporous silicon particles. Ball milling for 18 h and heat treatment at 1,050°C demonstrated to effectively convert oyster shell powders into Biphasic Calcium Phosphates (BCP) powders with a high content of β-TCP (Tricalcium Phosphate). Additionally, the anodization of silicon wafers was demonstrated to facilitate the presence of pore sizes ranging from 5 to 50 nm in the synthesized mesoporous silicon particles. Cytocompatibility tests of the calcium phosphate mixtures with mesoporous silicon particles with the murine pre-osteoblasts MC3T3-E1 evidenced very low/no cytotoxicity, as no or significant impact was shown in terms of cells proliferation of cellular morphology after 48 h, therefore deeming the formulated materials as cytocompatibility.

Another research hotspot highlighted in this Research Topic is the exploitation of bioactive materials to functionalise implants towards improving their osteoinductivity and/or preventing bacteria colonisation or infections. The review of the recent advances in antibacterial coatings for orthodontic appliances by Wang et al., identifies challenges associated with the presence of orthodontic appliances in orthodontic treatment, which lead to increased bacterial colonization and further enamel demineralization and periodontal diseases. The authors foresee that modifications in the form of coatings on the surface of orthodontic appliances can be an effective and practical approach to reducing bacterial proliferation and preventing relevant adverse effects. As highlighted by Wang et al., it is essential that orthodontic materials and coatings are evaluated in terms of 1) antibacterial, mechanical or physical properties at a relatively short period of time (few days and weeks) as well as long-term (up to months), 2) *in vitro* and *in vivo* studies to assess the biocompatibility, as the oral cavity is a complex environment with continuous changes in pH, saliva flow, and food chemicals; 3) friction reduction and advanced mechanical properties in order to establish the potential relationship between (anti) bacterial properties and other properties of orthodontic appliances.

In terms of bioactive materials exploited as a coating in metallic implants, TiO_2_ has attracted much attention because of its excellent antibacterial activity and biocompatibility. The antibacterial effect of TiO_2_ is mostly achieved through the process of photocatalysis in the ultraviolet (UV) region (<380 nm). Therefore, approaches of doping and surface modification allow TiO_2_ to exhibit catalytic activity within the visible light region, consequently improving the photocatalytic efficiency of TiO_2_. The research work by Kang et al. aimed at enhancing the osteogenic differentiation of bone mesenchymal stem cells while promoting antibacterial properties of dental implants by creating volcano-shaped microporous TiO_2_ coatings doped with copper (Cu) via micro-arc oxidation (MAO) on titanium substrates. For this, different mass ratios of Cu were studied in Cu-doped coating by changing the concentration of copper acetate in the electrolyte, obtaining that Cu-doped improves cell proliferation and facilitates osteogenic differentiation (during the period of 1–14 days of incubation) when compared to bare TiO_2_ coating. Moreover, the authors demonstrated that Cu-doped TiO_2_ coating possesses excellent antibacterial activity against *Staphylococcus aureus* and *Porphyromonas gingivalis* in short terms (24 h). Moreover, Tang et al. have presented an innovative approach to synthesize a titania (TiO_2_) coating on the surface of zirconia by infiltration in a suspension of zirconium oxychloride and titania for dense sintering with a further ultraviolet (UV) light treatment to enhance the biological inertness of zirconia. This study has evidenced promising cytocompatibility *in vitro* of MC3T3-E1 cells in terms of cell adhesion, proliferation, and osteogenic differentiation (ALP activity, mineralization, and upregulating osteogenic genes). Further, *in vivo* studies were performed on rat femurs to assess biocompatibility and host tissue response *in vivo*, demonstrating that UV-irradiated TiO_2_-modified zirconia implants maximized the promotion of osseointegration.

The exploitation of dual drug delivery platforms in bone tissue engineering has been presented in this Research Topic collection by Devi V. K. et al. as a promising approach to co-deliver supplementary bioactive compounds, such as distinct medicines and growth factors, for supporting enhanced bone regeneration. The delivery mode of the compounds from biomimetic platforms (e.g., porous 3D structures, microspheres, or films) can be modulated for a short or a prolonged time release by setting different parameters in the carrier platform (e.g., drug loading methodology, degree of crosslinking, morphology, pore size, porosity and the degradation rate of the material). In this line, Devi V. K. et al. have provided insights into the secret properties of marine-derived fucoidan (a sulphated polysaccharide derived from brown algae) for bone tissue engineering, and this biomaterial has shown promising outcomes in promoting the expression of type 1 collagen, osteocalcin, and BMP2 and aid in the mineral deposition. Moreover, the rheological properties of composites containing fucoidan have recently suggested potential as hydrogel and bio-inks for 3D bioprinting, which could lead to multiple applications in bone tissue engineering and drug delivery platforms in the field.

